# Estimation of the cool executive function using frontal electroencephalogram signals in first-episode schizophrenia patients

**DOI:** 10.1186/s12938-016-0282-y

**Published:** 2016-11-25

**Authors:** Yi Yu, Yun Zhao, Yajing Si, Qiongqiong Ren, Wu Ren, Changqin Jing, Hongxing Zhang

**Affiliations:** 1 Department of Biomedical Engineering, Xinxiang Medical University, Xinxiang, Henan People’s Republic of China; 2 Department of Psychology, Xinxiang Medical University, Xinxiang, Henan People’s Republic of China; 3 Department of Life Sciences and Technology, Xinxiang Medical University, Xinxiang, Henan People’s Republic of China

**Keywords:** Executive dysfunction, Complexity estimation, The cool executive function, Fractal dimension (FD)

## Abstract

**Background:**

In schizophrenia, executive dysfunction is the most critical cognitive impairment, and is associated with abnormal neural activities, especially in the frontal lobes. Complexity estimation using electroencephalogram (EEG) recording based on nonlinear dynamics and task performance tests have been widely used to estimate executive dysfunction in schizophrenia.

**Methods:**

The present study estimated the cool executive function based on fractal dimension (FD) values of EEG data recorded from first-episode schizophrenia patients and healthy controls during the performance of three cool executive function tasks, namely, the Trail Making Test-A (TMT-A), Trail Making Test-B (TMT-B), and Tower of Hanoi tasks.

**Results:**

The results show that the complexity of the frontal EEG signals that were measured using FD was different in first-episode schizophrenia patients during the manipulation of executive function. However, no differences between patients and controls were found in the FD values of the EEG data that was recorded during the performance of the Tower of Hanoi task.

**Conclusions:**

These results suggest that cool executive function exhibits little impairment in first-episode schizophrenia patients.

## Background

A variety of cognitive functions are consistently impaired in schizophrenia patients, among which executive dysfunction is the most critical cognitive impairment. Executive function is often seen as a significant high cognitive processing function that integrates flexible coordination of various processes to achieve a specific goal [1]. The prefrontal cortex plays an important role in executive control, and damage to the prefrontal cortex causes syndromes such as poor judgment, planning, and decision-making, which is characteristic of executive function degradation. A group of specific executive tests (the Wisconsin Card Sorting Test, Verbal Fluency Test, and Iowa Gambling Task) revealed deficits in patients with frontal lobe lesions compared with healthy controls [2]. Lovstad demonstrated that damage to the lateral prefrontal cortex (LPFC) particularly causes cognitive executive function deficits, while orbitofrontal cortex (OFC) injury is more strongly associated with self-reported dysexecutive symptoms in everyday living [3]. By comparing patients with lesions in different regions of the PFC, Ami found that damage to the left ventrolateral PFC impairs performance on the Stroop task and attention shifting tasks. In contrast, performance on the spatial search task depended on several PFC regions other than the left ventrolateral PFC [4].

Abnormal neuronal electrophysiological signals, especially electroencephalogram (EEG) signals, mainly appeared in the frontal cortex of schizophrenia patients [5]. Compared with healthy controls, patients with schizophrenia showed higher levels of delta and theta activity in the frontal region [6]. In drug-naive schizophrenia patients, gamma-band omega complexity was significantly higher, especially in the right frontal region [7]. Similarly, a higher complexity value was calculated at lower frequencies for drug-naive schizophrenia subjects compared with healthy controls using the multiscale entropy method [8]. In addition, EEG dimensional complexity in schizophrenia was lower than that in healthy controls [9]. In EEG studies, abnormal brain activity in schizophrenia patients can be detected using nonlinear analysis algorithms such as dimensional complexity [10], correlation dimension (D2) [11], Lempel_Ziv complexity (LZC) [12], approximate entropy (ApEn) [13], mutual information (MI) [14], and fractal dimension (FD) [15]. Among these algorithms, FD is applicable to nonlinear analysis of non-stationary and transient time series data like EEG signals [16], but FD has several limitations in stationary, noise-free, and long time series data. FD is related to entropy, which is directly related to the amount of signal information. Moreover, FD can be simply interpreted as the sinuosity, roughness, or the degree of irregularity of signals; thus, it is feasible to use FD to reveal the nonlinear information of EEG signals.

Since executive dysfunction is the most critical cognitive impairment, it is especially important to study the characteristics of EEG signals in schizophrenia patients during executive function tasks. Many executive function tasks have been widely studied in schizophrenia patients [17, 18]. Executive functions are divided into two types, i.e., a cool executive function that may be associated with relatively abstract and decontextualized tasks, and a hot executive function that uses a high degree of emotional involvement [19]. Cool executive function that is unrelated to emotional arousal is more objective and appropriate for studying executive function in schizophrenia patients because the emotional reaction of schizophrenia patients is often inconsistent with their inner experience [20]. At present, the research methods for cool executive function include search tasks, rule application tasks, conflict tasks, problem-solving tasks, and work memory tasks. However, studies of cool executive function using the analysis of EEG signals showed task-based and inconsistent results that cannot identify intrinsic defects in prefrontal function that contribute to poor performance in schizophrenia patients.

The present study diagnosed schizophrenia or evaluated the degree of damage to executive function in schizophrenia patients using cool executive tasks in complement with an evaluation of the complexity EEG data. We selected three cool executive tasks with different task difficulties, namely, the Trail Making Test-A (TMT-A) to estimate a more primitive consciousness movement rate, the Trail Making Test-B (TMT-B) to estimate quick visual search, visual space sorting, and cognitive set transfer functions, and the Tower of Hanoi task to estimate the ability to generate rules and make a plan based on those rules. Using an analysis of the FD of EEG signals in the frontal lobes of schizophrenia patients in comparison with healthy controls during the performance of different cool executive tasks, we intend to reveal new insight into the nature of schizophrenia.

## Methods

### Subjects and EEG recording

Seventeen first-episode schizophrenia patients who satisfied the DSM-IV diagnostic criteria-based structured clinical interview for DSM disorders (SCID) were enrolled from the Henan Psychiatric Hospital of China. The patients in this study were not treated with any medications; none had abused or depended on psychoactive substances, and none had a history of electroconvulsive therapy, mental retardation or dementia, other psychiatric or neurological diseases, or severe somatic diseases. The symptom severity was assessed using the positive and negative syndrome scale (PANSS). At the same time, 17 healthy controls matched for sex, age, and dominant side were also enrolled; none had psychiatric illness or a family history of psychiatric illness, other brain organic disease, or severe somatic diseases. The demographic and clinical characteristics of participants are shown in Table 1.

**Table 1:** Demographical and clinical situations about all participants

	Schizophrenia patients	Health controls
Participants	17	17
Male/female	10/7	10/7
Age	27.95 ± 7.02	24.84 ± 4.05
Course of the disease (month)	17.90 ± 7.12	–
Dominant hand	All right	All right
PANSS (total) score	≥60	–

The hospital ethics committee approved the study and all participants gave written informed consent. The data acquisition experiment was conducted from December 2014 to March 2015, and was performed in a quiet and light controlled room where the participants sat comfortably in a chair to perform three cool executive function tasks, i.e., the TMT-A, TMT-B, and the Tower of Hanoi task.

For the TMT-A task, subjects must quickly link numbers (1–25) in an increasing order with a pen, and in the process, the pen point must remain in contact with the paper. For the TMT-B task, subjects must quickly link numbers (1–13) and letters (A–M) according to an alternating sequence. The reaction time, which is the time subjects spent on every task, and the error number, which is calculated based on the errors in the numbers and letters that are linked, were used to evaluate the performance of the subjects in the two tasks. The Tower of Hanoi task used three tower bases that respectively had three wood blocks with different diameters. When moving the wood blocks, subjects should follow the following three rules: (1) one block can be moved per step; (2) the block must be placed on one of the three tower bases or the subjects’ hand; and (3) the lager blocks cannot be placed on top of the small blocks. In addition, the subjects need to move the blocks from the initial position to a target position. The performance of the subjects was evaluated based on the time to finish the task and their total operative steps.

During the experimental session, each subject performed three experimental tasks in a specific order from the TMT-A task, TMT-B task to the Tower of Hanoi task, according to the task difficulty degree. Moreover, between two trials, the subject had a rest for 10 min. The practice time varied among individuals and tasks, and lasted between 10 and 200 s. Meanwhile, the EEG recording time for the subject was equal to his/her practice time, in order to estimate the FD complexity of EEG data during the whole task period. Therefore, the length of EEG data for estimating FD values also varied among individuals and tasks. In addition, before performing the tests in each task, the subjects had to clearly understand the tasks and were not provide any training to avoid the potential influence for reaction time due to task proficiency.

According to the 10–20 international system, the EEG data were recorded though 14 electrodes (FP1, FPz, FP2, AF3, AF4, F7, F5, F3, F1, Fz, F2, F4, F6, and F8) that were mounted on the scalp with a 64-channel EEG cap, and 1000 Hz was sampled with a low pass filter of 125 Hz. The recorded data size for each subject was dependent on the time spent on the executive function tasks.

### Modified wavelet packet threshold applied to electrooculography (EOG) artifact removal

For EEG analysis, a critical problem is that signals are susceptible to physiological artifacts in data recording [21]. In the present study, EEG data that was recorded from the frontal lobes was severely contaminated by EOG artifacts. Therefore, removing EOG artifacts is essential for further EEG analysis. For better analysis of the EEG signals, the sampling frequency was reduced to 128 Hz to reduce data redundancy. The useful EEG data is often present in the low frequency band; thus, our sampling frequency ensured the retention of the useful EEG data. The Independent Component Analysis (ICA) algorithm is often used to separate EOG artifacts from EEG data [22, 23]. However, as a batch algorithm, ICA must be performed on all of the data with an adequate number of signals, and its computation is extremely complex and time-consuming. In contrast, the wavelet threshold algorithm can remove EOG artifacts from single-channel data with the advantage of multi-resolution analysis of wavelet transforms [24]. Nevertheless, the traditional wavelet threshold method using some soft and hard threshold functions for threshold wavelet coefficients cannot be uniformly compressed in positive and negative directions. Therefore, we used a modified adaptive threshold technique to process wavelet packet coefficients decomposed from the schizophrenia EEG signals in the frontal lobe in the first two nodes of the third layer, which includes the EOG artifacts.

1 $$Thr_{(i,j)} = \sigma_{(i,j)} *\sqrt {2\log_{e} (N_{(i,j)} \log_{10} (N_{(i,j)} ))} \;$$

where, *Thr*_(*i*,*j*)_ is the modified adaptive wavelet packet threshold in the j-th node of the i-th layer, *N*_(*i*,*j*)_ is the length of the wavelet packet coefficients, and *σ*_(*i*,*j*)_ is the correction factor.

2 $$\begin{aligned} \sigma _{{\left( {i,j} \right)}} &= median\left( {\left| {C_{{\left( {i,j} \right)}} } \right|} \right)*c \\ C_{{\left( {i,j} \right)}} &= \left\{ {\begin{array}{*{20}l} {C_{{\left( {i,j} \right)}} } & \quad {\left| {C_{{\left( {i,j} \right)}} - mean\left( {C_{{\left( {i,j} \right)}} } \right)} \right| < Thr_{{\left( {i,j} \right)}} } \\ {mean\left( {C_{{\left( {i,j} \right)}} } \right)} & \quad {\left| {C_{{\left( {i,j} \right)}} - mean\left( {C_{{\left( {i,j} \right)}} } \right)} \right| > Thr_{{\left( {i,j} \right)}} } \\ \end{array} } \right. \end{aligned}$$

where, *median*(|*C*_(*i*,*j*)_|) is the median of the absolute value of wavelet coefficients, *c* is the empirical factor, set to 0.6, and is the modulated wavelet packet coefficient that overcomes the shortcoming that wavelet packet coefficients in traditional hard and soft threshold algorithms cannot be uniformly compressed in positive and negative directions.

### FD

Based on fractal geometry, a measuring tool for complex systems, FD reflects the irregularity of complex shapes, which is extremely intensive in data scaling, especially biological data [25]. With the advantage of measuring the self-similarity of signals, FD has been widely used in analyzing the complexity of nonlinear signals. Many algorithms have been used to calculate FD, such as Petrosian, Katz, Higuchi, and box-counting. In the present study, the box-counting algorithm is applied to the FD estimation of the EEG data.

The box-counting algorithm calculates FD as follows: [26]

3 $${\text{D}} = \mathop {\lim }\limits_{\varepsilon \to 0} \;[\log_{2} (N(\varepsilon ))/\log_{2} (1/\varepsilon )]\;$$

where, *ɛ* is the side length of boxes, *N*(*ɛ*) is the number of contained boxes computed from the difference between the maximum and minimum amplitudes of the data divided by the changed side length *ɛ*, as follows

4 $$\begin{array}{l} {n_{\varepsilon } \left( i \right) = {{\left( {\text{max} \left( {x_{\varepsilon } } \right){ - }\text{min} \left( {x_{\varepsilon } } \right)} \right)} /{\varepsilon_{i} ,}}} \\ \quad \quad \quad \quad {\varepsilon \in 2^{k} \left| {k = 1,2, \ldots \log_{2} \left( L \right){ - }1} \right.} \\ {N\left( \varepsilon \right) = \sum\limits_{i} {n_{\varepsilon } \left( i \right)} } \\ \end{array}$$

where, *N*(*ɛ*) is the sum of contained boxes, *x*_*ɛ*_ is the time series of data with length L. For calculating the FD, according to the least-square procedure, the slope of log_2_(*N*(*ɛ*)) versus log_2_(1/*ɛ*) is obtained.

### Statistical analysis

Group differences between the first-episode schizophrenia patients and healthy controls were analyzed by using independent sample t test using the MATLAB statistical toolbox. A repeated-measures analysis of variance (ANOVA) on one factor was conducted to determine whether there was a statistical significance between two groups in terms of FD values during all different experimental tasks. The independent variables included a between-subjects factor, the groups as the first-episode schizophrenia patients and healthy controls, and within-subject variable, three different experimental tasks as the TMT-A, TMT-B, and the Tower of Hanoi task. Therefore, the groups and the experimental tasks were the main variables. The dependent variable was the FD. Hence, using a repeated measure ANOVA in the nonlinearity measure has been investigated statistically as a result of interaction between the participant groups and experimental tasks. Before ANOVA, the homogeneity of the slopes between the groups was assessed with a Levene test. If the slopes were homogeneous, ANOVA was performed.

## Results

For preprocessing of EEG data in the frontal lobes, it is important to remove the EOG artifacts. Therefore, a wavelet packet transform (WPT) was used to decompose each EEG signal at level three using Daubechies wavelets of order four, which are more adaptive for the detection of changes in the EEG signals.

**Fig. 1:**
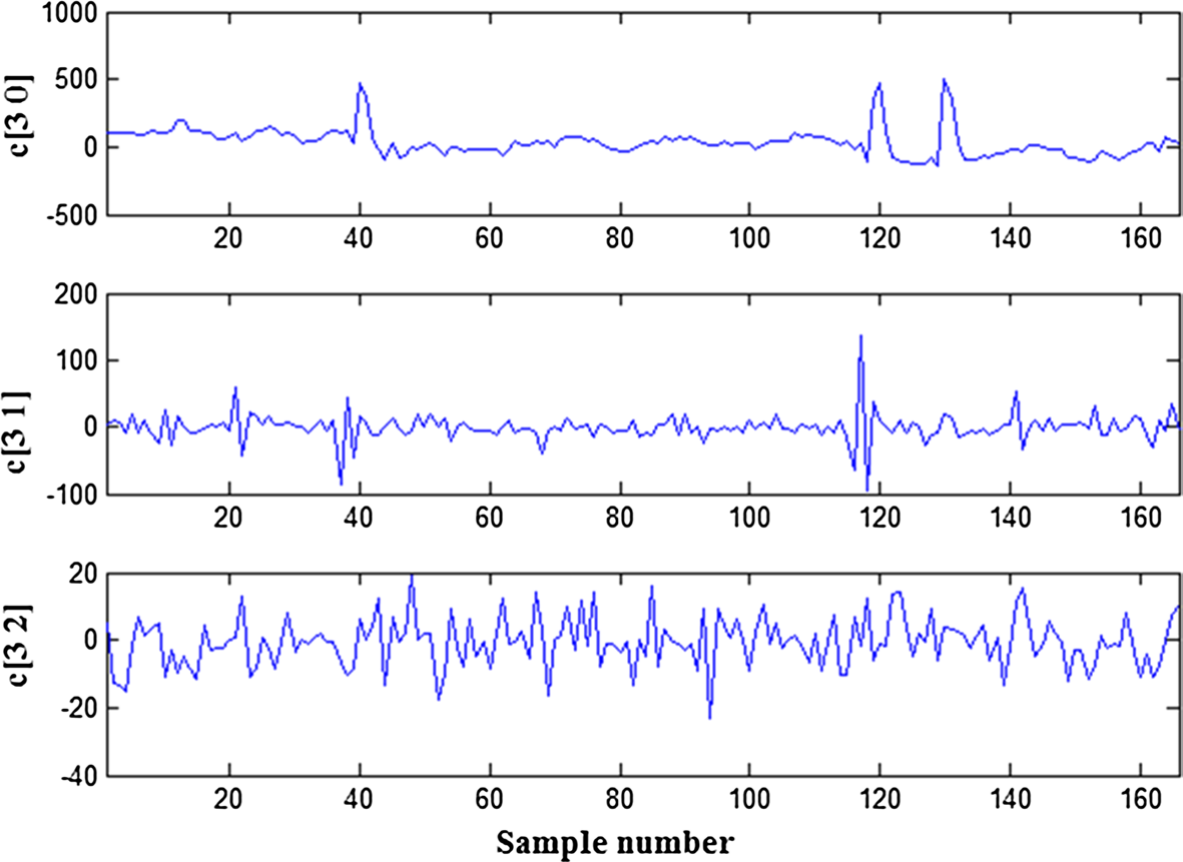
The wavelet packet coefficients in the third layer of EEG signal in a patient PF1 channel

**Fig. 2:**
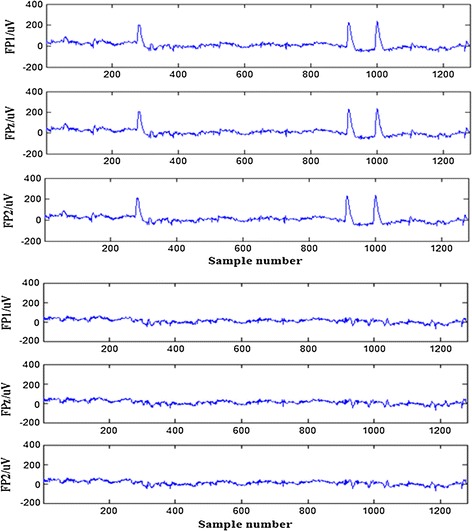
The comparison between the EEG signals in a patient channels (FP1, FPz, FP2) with and without EOG artifacts

Figure 1 shows the EOG artifacts focused on the wavelet packet coefficients in the first two nodes of the third layer, which were processed using a modified adaptive threshold technique. Finally, the EEG signals with the EOG artifacts removed were reconstructed using an inverse wavelet packet transform (IWPT). Figure 2 shows a comparison between the EEG signals in patient channels (FP1, FPz, FP2) with and without the EOG artifacts.

**Fig. 3:**
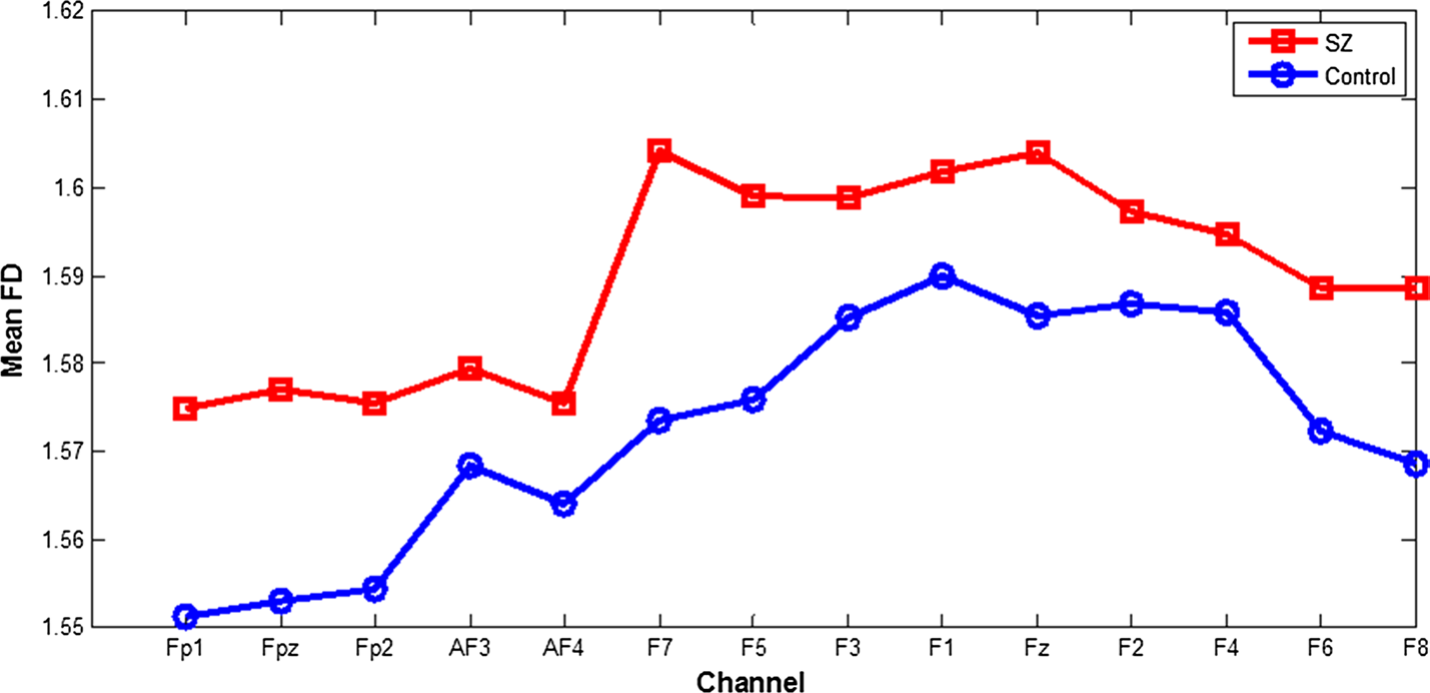
Comparison between the means of FD values calculated from EEG signals in frontal lobes during manipulation of the TMT-A task between patients and control

**Fig. 4:**
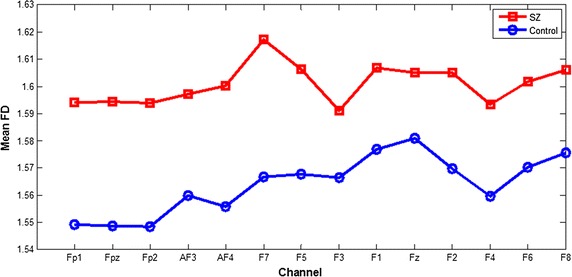
Comparison between the means of FD values calculated from EEG signals in frontal lobes during manipulation of the TMT-B task between patients and controls

**Fig. 5:**
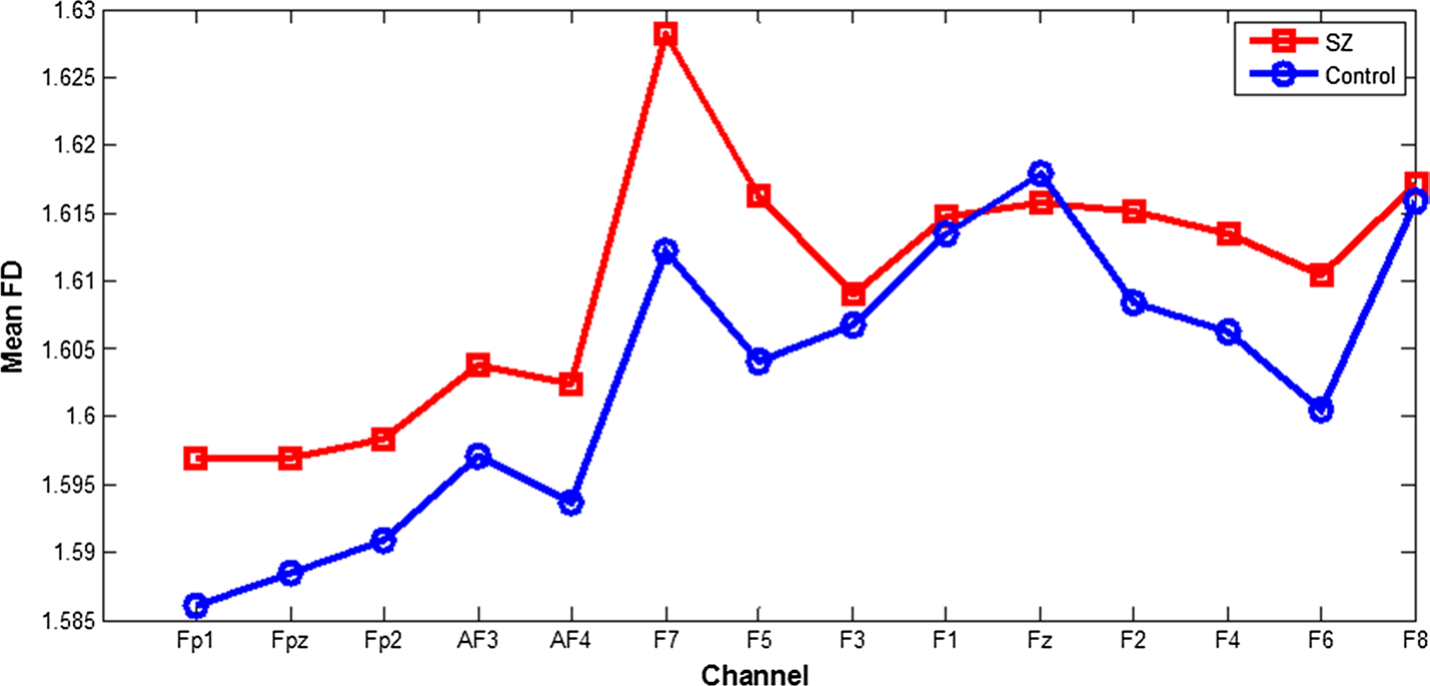
Scatter plot with FD values of in F7 channel during the performance of TMT-A task, TMT-B task and the Tower of Hanoi tasks respectively

The FD values and the standard deviation values calculated from the preprocessed EEG signals of first-episode schizophrenia patients and healthy controls during the performance of three cool executive tasks are respectively shown in Tables 2, 3, and 4. Here, it was revealed that the FD values of the patients were higher than those of the healthy controls during the performance of TMT-A and TMT-B tasks. A t test showed that this difference was statistically significant for most channels of TMT-B task, but for only two channels (F5, F7). Comparisons between the means of the FD values calculated from the frontal lobe EEG signals during the TMT-A/B tasks in patients and controls are respectively shown in Figs. 3 and 4. The largest differences were found in the left frontal lobe (F7) during the performance of both TMT-A/B tasks, and the corresponding scatterplots of both patients and controls in channel F7 were shown in Fig. 5.

**Table 2:** Comparison of the FD values calculated from EEG signals in frontal lobes during manipulation of the TMT-A task between patients and the controls (mean ± SD)

Channels	The patients	The controls	P value
FP1	1.5749 ± 0.0422	1.5513 ± 0.0389	0.100
FPz	1.5771 ± 0.0427	1.5531 ± 0.0399	0.101
FP2	1.5755 ± 0.0463	1.5543 ± 0.0405	0.166
AF3	1.5796 ± 0.0465	1.5685 ± 0.0315	0.421
AF4	1.5755 ± 0.0463	1.5641 ± 0.0321	0.413
F7	1.6042 ± 0.0315	1.5735 ± 0.0318	0.008*
F5	1.5990 ± 0.0293	1.5760 ± 0.0312	0.034*
F3	1.5988 ± 0.0370	1.5851 ± 0.0377	0.293
F1	1.6018 ± 0.0343	1.5899 ± 0.0239	0.249
Fz	1.6040 ± 0.0336	1.5853 ± 0.0292	0.094
F2	1.5972 ± 0.0425	1.5867 ± 0.0277	0.397
F4	1.5946 ± 0.0473	1.5858 ± 0.0310	0.524
F6	1.5885 ± 0.0471	1.5724 ± 0.0248	0.221
F8	1.5886 ± 0.0469	1.5686 ± 0.0254	0.132

* Statistically significant difference

**Table 3:** Comparison of the FD values calculated from EEG signals in frontal lobes during manipulation of the TMT-B task between patients and the controls (mean ± SD)

Channels	The patients (±SD)	The controls (±SD)	P value
FP1	1.5942 ± 0.0342	1.5493 ± 0.0371	0.001*
FPz	1.5943 ± 0.0357	1.5486 ± 0.0364	0.001*
FP2	1.5939 ± 0.0343	1.5484 ± 0.0343	0.001*
AF3	1.5971 ± 0.0354	1.5598 ± 0.0349	0.004*
AF4	1.6002 ± 0.0331	1.5575 ± 0.0332	0.000*
F7	1.6171 ± 0.0404	1.5666 ± 0.0492	0.003*
F5	1.6063 ± 0.0482	1.5677 ± 0.0410	0.017*
F3	1.5911 ± 0.0563	1.5664 ± 0.0360	0.138
F1	1.6068 ± 0.0357	1.5768 ± 0.0315	0.014*
Fz	1.6051 ± 0.0337	1.5811 ± 0.0272	0.029*
F2	1.6049 ± 0.0336	1.5697 ± 0.0381	0.007*
F4	1.5934 ± 0.0488	1.5598 ± 0.0523	0.062
F6	1.6018 ± 0.0367	1.5702 ± 0.0281	0.020*
F8	1.6060 ± 0.0394	1.5757 ± 0.0265	0.013*

* Statistically significant difference

**Table 4:** Comparison of the FD values calculated from EEG signals in frontal lobes during manipulation of the Tower of Hanoi task between patients and the controls (mean ± SD)

Channels	The patients	The controls	P value
FP1	1.5969 ± 0.0392	1.5861 ± 0.0268	0.352
FPz	1.5970 ± 0.0399	1.5885 ± 0.0272	0.471
FP2	1.5984 ± 0.0384	1.5909 ± 0.0273	0.518
AF3	1.6038 ± 0.0398	1.5971 ± 0.0275	0.570
AF4	1.6024 ± 0.0464	1.5937 ± 0.0277	0.511
F7	1.6282 ± 0.0312	1.6121 ± 0.0309	0.142
F5	1.6162 ± 0.0482	1.6040 ± 0.0357	0.409
F3	1.6091 ± 0.0465	1.6067 ± 0.0339	0.867
F1	1.6148 ± 0.0362	1.6134 ± 0.0305	0.907
Fz	1.6158 ± 0.0411	1.6179 ± 0.0290	0.860
F2	1.6152 ± 0.0368	1.6084 ± 0.0492	0.652
F4	1.6134 ± 0.0400	1.6062 ± 0.0385	0.596
F6	1.6104 ± 0.0441	1.6006 ± 0.0405	0.505
F8	1.6171 ± 0.0418	1.6159 ± 0.0263	0.916

**Fig. 6:**
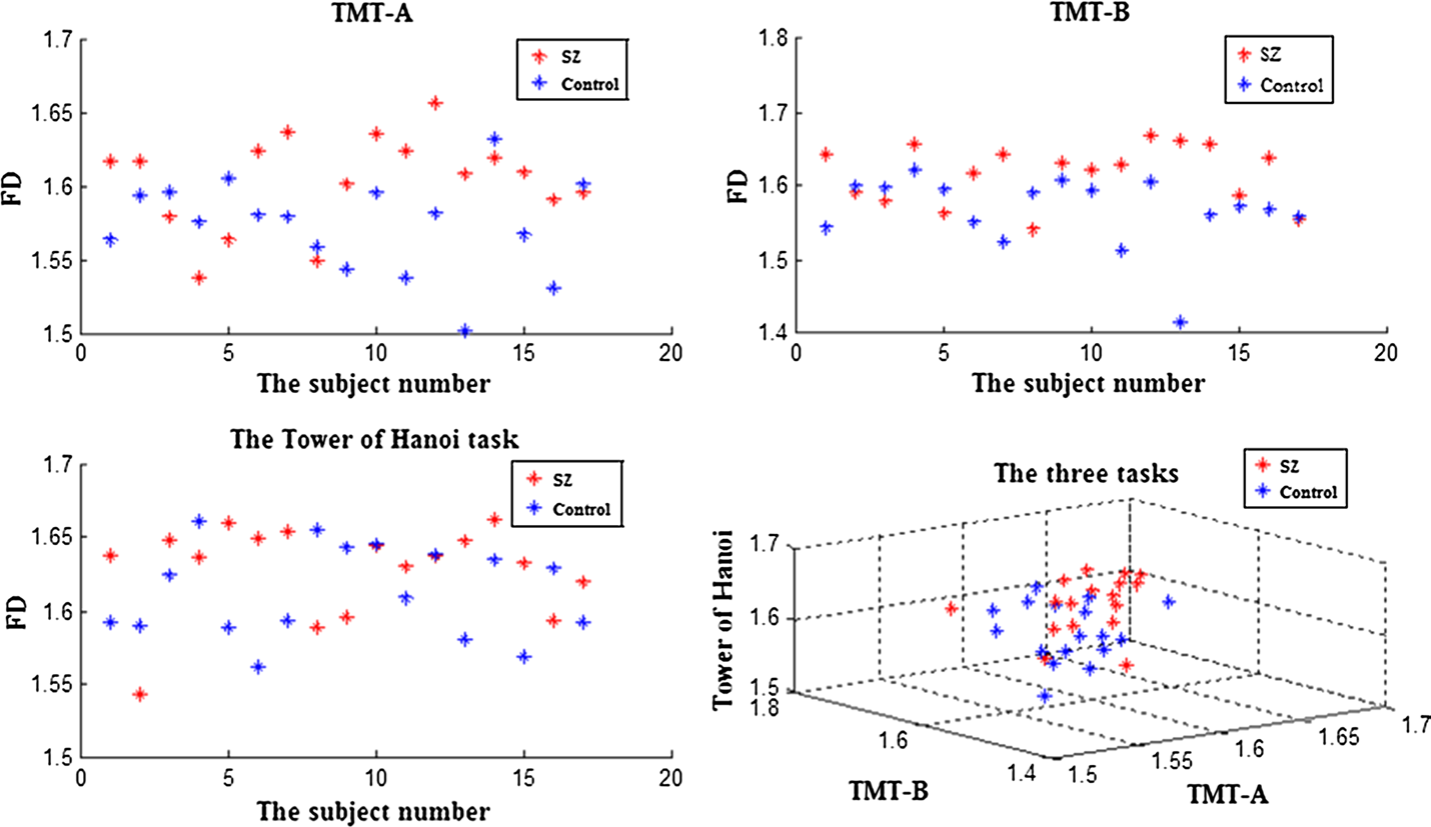
Comparison between the means of FD values calculated from EEG signals in frontal lobes under the Tower of Hanoi task between patients and controls

Table 4 shows that the FD values in patients and controls were approximately equal in each channel during the performance of the Tower of Hanoi task. The t test found no statistically significant difference in each channel. Figure 6 shows the average FD complexity of the patients and controls in each channel.

Different results obtained from a comparison between the patients and controls were generated for the three executive tasks of different difficulties. Therefore, the FD values of the EEG signals for the three different executive tasks were analyzed using a repeated-measure ANOVA with one between-subjects (patient versus control group) and one within-subjects (experimental tasks) variable. No significant interactions between the two variables were found for all channels in FD values. There was a significant main effect of group for FD values in all channels. However, no significant main effect of the experimental tasks was found for all channels in FD values, as shown in Table 5.

**Table 5:** A repeated-measures ANOVA with one between-subjects (patient versus control group) and one within-subjects (experimental tasks) variable

Channel	A repeated-measures ANOVA					
	The main effect of group		The main effect of group		Interaction	
	F	P	F	P	F	P
FP1	26.02	0.0000	0.6	0.5484	0.92	0.4031
FPz	26.26	0.0000	0.32	0.7247	0.93	0.3965
FP2	25.17	0.0000	0.31	0.7337	1.19	0.309
AF3	15.45	0.0002	0.17	0.8465	1.44	0.2414
AF4	22.52	0.0000	0.6	0.5521	2.45	0.0913
F7	29.15	0.0000	0.06	0.9414	0.66	0.5205
F5	17.4	0.0001	0.00	0.9982	0.42	0.6575
F3	5.75	0.0184	1.01	0.3668	0.17	0.8407
F1	13.98	0.0003	0.18	0.8377	0.88	0.4161
Fz	13.21	0.0005	0.03	0.9703	0.09	0.9176
F2	14.44	0.0003	0.19	0.8272	1.34	0.2662
F4	7.32	0.0081	0.94	0.3941	0.78	0.4633
F6	12.63	0.0006	0.25	0.7788	0.48	0.6215
F8	15.02	0.0002	1.38	0.2575	0.25	0.7822

In this paper, we have made the scatterplots of FD values in F7 channel both patients and controls during the performance of TMT-A, TMT-B and the Tower of Hanoi tasks respectively in Fig. 5. Meanwhile, with FD values of in F7 channel during the performance of TMT-A task, TMT-B task and the Tower of Hanoi tasks respectively as X axis, Y axis and Z axis, a three-dimension scatterplot was drawn in Fig. 5. It revealed that during the performance of TMT-B task, the data points of both patients and controls were more concentrated and only minority data points overlapped each other between the two groups. However, during the performance of TMT-A task and the Tower of Hanoi task, the data points of both patients and controls were more scattered and overlapped each other between the two groups, especially for the Tower of Hanoi task. Those results were consistent with the results of statistical analysis for FD values between two groups in Tables 2, 3 and 4. Meanwhile, the three-dimension scatterplot revealed the results that with all the FD values of three tasks as the features, the data points had less overlap each other between the two groups, which is consistent with a significant main effect of group for FD values in all channels using a repeated-measure ANOVA with one between-subjects (patient versus control group) and one within-subjects (experimental tasks) variable.

To better analyze the difference in the FD values between the patients and controls, we recorded the reaction time and error number of subjects while they solved the three tasks. The statistical analysis of these data are shown in Table 6, indicating that compared with the controls, patients spent more time on the three tasks, with statistical differences based on a t test (*P* = 0.013, 0.001, and 0.001). Moreover, the error number of patients during the performance of the TMT-B task is higher than the controls, with statistical difference based on a nonparametric independent sample t test (*P* = 0.015). Nevertheless, no statistical difference in the error number was found between the patients and controls during the performance of the TMT-A task and the Tower of Hanoi task (*P* = 0.333 and 0.874, respectively).

**Table 6:** Comparison of the task performance between patients and controls (mean ± SD)

	The Tower of Hanoi		TMT-A		TMT-B	
	Reaction time	Operative steps	Reaction time	Error number	Reaction time	Error number
Controls	34.12 ± 18.55	9.32 ± 3.132	36.76 ± 14.24	0.24 ± 0.59	75.68 ± 25.17	0.28 ± 0.792
Patients	60.76 ± 42.39*	10.19 ± 3.71	60.29 ± 18.92*	0.24 ± 0.70	132.52 ± 64.68*	1.57 ± 2.52*

* Statistically significant difference

## Discussion

In recent years, complexity estimators have been increasingly applied to analyze the EEG data of schizophrenia patients. However, both increased and decreased values for complexity estimators have been reported, which might be due to medication effects, age effects, or different algorithms of complexity estimators [27]. Considering these factors, the subjects selected for this study were first-episode schizophrenia patients who were drug-naïve and aged 27.95 ± 7.02 years. In addition, we used an FD algorithm as a complexity estimator, with the advantage of measuring the self-similarity of the signals. In schizophrenia, executive dysfunction is a critical impairment and is associated with abnormal neuronal electrophysiological activities of the prefrontal areas [28]. The EEG signals analyzed in the present study were recorded in the frontal lobes of selected patients during the performance of three cool executive function tasks to explore executive function impairment in first-episode schizophrenia patients.

Artifact removal from the EEG signals before analysis is extremely important for further EEG analysis. The EEG data in the present study were severely contaminated by EOG artifacts. Therefore, the first task was to remove the EOG artifacts from the EEG data. In previous studies, many methods have been proposed to remove EOG artifacts, such as principal component analysis (PCA), independent component analysis (ICA), and wavelet transforms (WT). In the present study, we used a modified adaptive wavelet packet threshold technique to remove EOG artifacts from single channel data, with the advantage of multi-resolution analysis; Fig. 2 shows that the EOG artifacts have been removed from the EEG signals in the frontal lobe channels.

Complexity estimators have been widely used for EEG analysis of schizophrenia patients, and these estimators have performed well. The present study used an FD algorithm to estimate the complexity of the EEG data in the frontal lobes of first-episode schizophrenia patients and healthy controls during the performance of three cool executive function tasks. In the present study, patient FD values that were calculated from the EEG data during the performance of the TMT-A and TMT-B tasks were higher than those of the healthy controls, and this difference was statistically significant for most channels for TMT-B task, but only two channels (F7 and F5) for TMT-A task. This result is consistent with previous research showing that increased irregularity in neurophysiological activity of schizophrenia patients generated EEG data with increased complexity, especially in the frontal lobes [27]. Moreover, this result is consistent with the fact that patients that spent more time on the TMT-A and TMT-B tasks than the controls, with a statistical difference in a t test (respectively *P* = 0.013 and 0.001). In addition, the error number for patients during the performance of the TMT-B task was higher than that in the controls, with a statistical difference in a nonparametric independent sample t test (*P* = 0.015). Therefore, we estimated that cool executive function exhibits some deficits in first-episode schizophrenia patients. However, there was no difference in the FD values calculated from the EEG data during the performance of the Tower of Hanoi task between the patients and controls; this finding is consistent with the results showing no statistically significant difference in operative steps for the Tower of Hanoi task between the patients and controls, which may be related to less damage to planning and working memory ability in first-episode schizophrenia patients. Moreover, we estimated that the level of difficulty of executive tasks may strongly influence the complexity of the EEG data; but this finding was not consistent with the results that no significant main effect of the experimental tasks was found for all channels in FD values, which is consistent with a study showing that the level of task difficulty had little influence on patient performance [29]. Meanwhile, the scatterplot of FD values in F7 channel during TMT-B task were more concentrated and only minority data points overlapped each other between the two groups, but not for the other two tasks, were consistent with the results of statistical analysis for FD values between two groups, and the three-dimension scatterplot showed the same results of a significant main effect of group for FD values in all channels using a repeated-measure ANOVA, which cannot better reveal the difference of FD values between the two groups. Therefore, in future study, we should select the more appropriate cool executive tasks and increase the number of subjects to clearly discriminate the complexity of EEG signals between tasks. Moreover, we found small differences in the FD values between patients and controls, which is similar to a previous study by Akar [30], and may be related to less damage to executive function in first-episode schizophrenia patients. However, the difference in the FD values between normal and schizophrenia subjects is in the opposite direction in these two studies. The conflict may be due to the patients’ clinical status, symptom severity, medication, or age status. Moreover, a previous study by Akar focused on the effect of noise on the complexity of the EEG data, while our study focused on the effect of the task status on the complexity of the EEG data. Therefore, more complexity measures should be used to estimate the complexity of EEG signals in schizophrenia patients so that larger differences may be found between the patients and controls, which can be applied to clinical diagnosis for schizophrenia and even other psychiatric disorder in the future.

## Conclusion

Our results demonstrate that the complexity of frontal EEG signals measured using FD was different in first-episode schizophrenia patients during the manipulation of executive function. Moreover, cool executive function exhibited little damage in first-episode schizophrenia patients. However, our study has some limitations. First, the EEG data in our study were recorded from the frontal lobes, but not any other brain areas. Second, the relevant EEG data of the first-episode schizophrenia patients should be recorded for analysis after a period of medication treatment. Therefore, in future studies, it will be informative to estimate cool executive function in first-episode schizophrenia patients with and without medication treatment using nonlinear analysis of EEG data from several brain areas. In this manner, a medical standard may be developed to diagnose schizophrenia or the degree of damage to executive function in schizophrenia patients.
